# “We Wait”—The Impact of Character Responsiveness and Self Embodiment on Presence and Interest in an Immersive News Experience

**DOI:** 10.3389/frobt.2018.00112

**Published:** 2018-10-04

**Authors:** Anthony Steed, Ye Pan, Zillah Watson, Mel Slater

**Affiliations:** ^1^Department of Computer Science, University College London, London, United Kingdom; ^2^BBC Research & Development/BBC Virtual Reality Hub, London, United Kingdom; ^3^Clinical Psychology and Psychobiology, University of Barcelona, Barcelona, Spain; ^4^Institució Catalana de Recerca i Estudis Avançats (ICREA), Barcelona, Spain

**Keywords:** immersive journalism, virtual reality, refugee crisis, presence, place illusion, plausibility, embodiment, body ownership

## Abstract

A virtual reality scenario called “We Wait” gives people an immersive experience of the plight of refugees waiting to be picked up by a boat on a shore in Turkey to be illegally taken to Europe, crossing a dangerous stretch of sea. This was based on BBC news reporting of the refugee situation, but deliberately depicted as an animation with cartoon-like characters representing the refugees. Of interest was the level of presence that might be experienced by participants and the extent to which the scenario might prompt participants to follow-up further information about the refugee crisis. By presence we refer to both Place Illusion, the illusion of being in the rendered space, and Plausibility, the illusion that the unfolding events were really happening. The follow-up was assessed by whether and when participants accessed a web page that contained further information about the refugee crisis after the experiment. Two factors were considered in a balanced between-groups design with 32 participants. The Responsiveness factor was either “None” or “Look at.” In the first the virtual characters in the scenario never responded to actions of the participant, and in the second they would occasionally look at the participant after the participant looked at them. The second factor was Embodiment, which was either “No Body” or “Body.” In the No Body condition participants had no virtual body, and in the Body condition they would see a virtual body spatially congruent with their own if they looked down toward themselves. The virtual body was animated by the head tracking move the upper body. The results showed that the major factor positively contributing to presence was Responsiveness (“Look at”), and that Embodiment (“Body”) may have contributed but to a lesser extent. There were important differences between men and woman in the degree of follow-up, with men more likely to do so than women. The experiment shows that adding in some simple responses in an immersive journalism scenario, where the characters acknowledge the presence of the participant through gaze, can enhance the degree of presence felt by the participants.

## Introduction

Immersive Virtual Reality (VR) for the portrayal of current affairs stories was first introduced by de la Peña et al. (2010) where participants experienced from a first person perspective a situation based on the interrogation of a Guantánamo Bay prisoner. The scenario was delivered using a head-tracked wide field of view head-mounted display (HMD), with real-time motion capture of the movements of the participant that were mapped to a life-sized virtual body that apparently substituted the person's real body seen from their first person perspective (1PP). In other words when participants would look down toward their actual body while in the VR, they would see the virtual body instead. The first novel aspect of this was that although participants were seated comfortably in a chair, they experienced the virtual reality as if they were standing in a stress position, alone in an empty cell, but where they could hear an interrogation in the cell next door. Hence their virtual body posture did not match their real posture, but the participants in the case study experienced discomfort, and anxiety due to the harsh interview techniques that they could hear^1^. The second novel aspect was that the piece used parts of the transcript of the actual interrogation of a prisoner that was heard as if from the adjacent cell. The approach was referred to as “immersive journalism” a term, which although widely used, is not quite appropriate—since is not the journalism that is “immersive” but the delivery of its results. The basic premise of “immersive journalism” is that people can read news stories, or watch them on TV, but personal and direct experience can never be achieved that way—whereas VR has the possibility to provide this.

In particular VR can deliver four related illusions that together go beyond just 3D surrounding content. The first two are components of what is usually called *presence* (Held and Durlach, 1992; Sheridan, 1992; Sanchez-Vives and Slater, 2005). We refer to “Place Illusion” (PI) as the illusion of being in the place depicted by the VR, irrespective of what might be depicted as happening there. This is a perceptual illusion, based on the extent to which the system delivers natural sensorimotor contingencies—that is to be able to use our whole bodies for perception in the normal way (Slater, 2009). We use this term rather than just “presence” since the latter has been confounded with many other attributes of an experience, such as the degree of interest, engagement, “flow” and so on. While all of these other attributes are important, we distinguish them from PI, which refers solely to the illusion of being in the place. The “Plausibility Illusion” (Psi) is the illusion that the events depicted in the VR are really occurring (Slater, 2009). This can be facilitated by (i) the virtual world responding to participant actions (ii) contingent events that refer personally to the participant (iii) the extent to which the portrayal of the virtual world and its events conforms to expectations where this is applicable (Bergstrom et al., 2017) and maintains internal consistency (Skarbez et al., 2017). Body ownership is the third illusion, referring to the extent that a virtual self-representation within the VR is illusorily experienced as the participant's own body (Slater et al., 2010b; Blanke et al., 2015). The fourth illusion, closely related to body ownership is agency, that actions of the virtual body are attributed to the self (Haggard and Chambon, 2012), which may be veridical agency (where the virtual body moves synchronously and in correlation with real body movements) or illusory agency, where actions of the virtual body are self-attributed even when its actions were not those of the participant (Wegner et al., 2004; Banakou and Slater, 2014, 2017). In the case of each illusion, of course participants know that they are illusions, and yet nevertheless their responses at many levels are as if these were real.

The research we describe in this paper started from an already existing VR news scenario known as “We Wait.”^2^ This depicts a situation on a shore in Turkey amongst a group of refugees, waiting for a boat to arrive to take them to Europe. Our goal was to explore whether PI, Psi and body ownership and subsequent follow-up of information about the refugee crisis were impacted by two factors related to the design of the virtual environment. The two factors were “Responsiveness” and “Embodiment.” The two levels of Responsiveness were “None” and “Look at.” In the first the virtual characters did not respond or look at the participant. In the second virtual refugees occasionally looked at the participant when the participant looked toward them. The Embodiment factor consisted of “No Body” or “Body.” In the first if the participant looked down toward themselves they would be invisible, and in the second they would see a virtual body substituting their own. The body was animated only with respect to head position, no hand-tracking was done, but nonetheless it could be seen as substituting the participant's own body from first person perspective. We used questionnaires to assess the level of PI, Psi and body ownership. In addition, after the experience participants were given a link to a web page where they could follow-up to find out more information about the refugee crisis. We were interested in whether and how many times, and how long after the experience the participants would look at this web page, and whether this was influenced by the factors (Responsiveness and Embodiment).

Our hypothesis was that the responsiveness of the virtual characters towards the participant, and the participant having a virtual body would lead to greater reported levels of PI, Psi and body ownership. Moreover, we were interested to discover how these factors influenced whether participants engaged in follow-up indicated by accessing the web page.

## Background

### Immersive journalism

Virtual Reality representation of news and current events typically provides a passive experience where the participant simply observes an unfolding scenario/story, albeit in a 360 degree surrounding world. It is usually video based, sometimes model based, and with stereo vision. Although there has been a long tradition of using VR for narrative, for example, (Pausch et al., 1996), since the work by (de la Peña et al., 2010) there has been a growing interest in and examples of immersive journalism. Nonny de la Peña's group developed “Hunger in Los Angeles” about events on a food line in Los Angeles which was exhibited at the Sundance Film Festival in 2012. The 2014 World Economic Forum included her “Project Syria” about a bomb explosion in a Syrian town. “One Dark Night” depicted the shooting of teenager Travyon Martin, and “Kiya” about murder in the context of domestic violence. All of these were based on real events, and combined graphics rendering of the scenarios with recorded sounds and other information from the depicted events.

Early in 2014, interest in using 360 video for HMD viewing to create immersive journalism began to grow. For example, Louis Jebb and Edward Miller produced a 360 film of a protest in Hong Kong in 2014 (Hong Kong Unrest). In 2015 the UN sponsored “Clouds over Sidra,” a VR documentary film about a child refugee in the Syrian war, which was created by Gabo Arora and Chris Milk.

Chris Milk's description of VR as “the ultimate empathy machine” in a Ted Talk in April 2015^3^ further fuelled interest in VR's potential for news. Empathy was one of the promises of VR explored by the Tow Report *Virtual Reality Journalism*: “a core question is whether virtual reality can provide similar feelings of empathy and compassion to real-life experiences” (Aronson-Rath et al., 2016).

Since then, many news organizations and broadcasters including The New York Times, The Guardian and the BBC have developed a number of other 360 degree VR news stories or documentaries. Overall the topic has generated a great deal of interest (Doyle et al., 2016; Slater and Sanchez-Vives, 2016; Watson, 2016).

In “We Wait,” the BBC and Aardman Interactive, experimented with using computer graphics-based VR to put the viewer in a place that few foreign reporters could risk—on a boat with a group of refugees crossing the Mediterranean or waiting on a beach for the boat to appear. The purpose was to create the feeling that the participant was part of the unfolding events. Though scripted, it was based on BBC news coverage of the migrant crisis in 2015. “We Wait” was premiered at the Sheffield Documentary Festival in 2016^4^. It won the Broadcast Digital Award for VR in 2017^5^.

**Figure 1:**
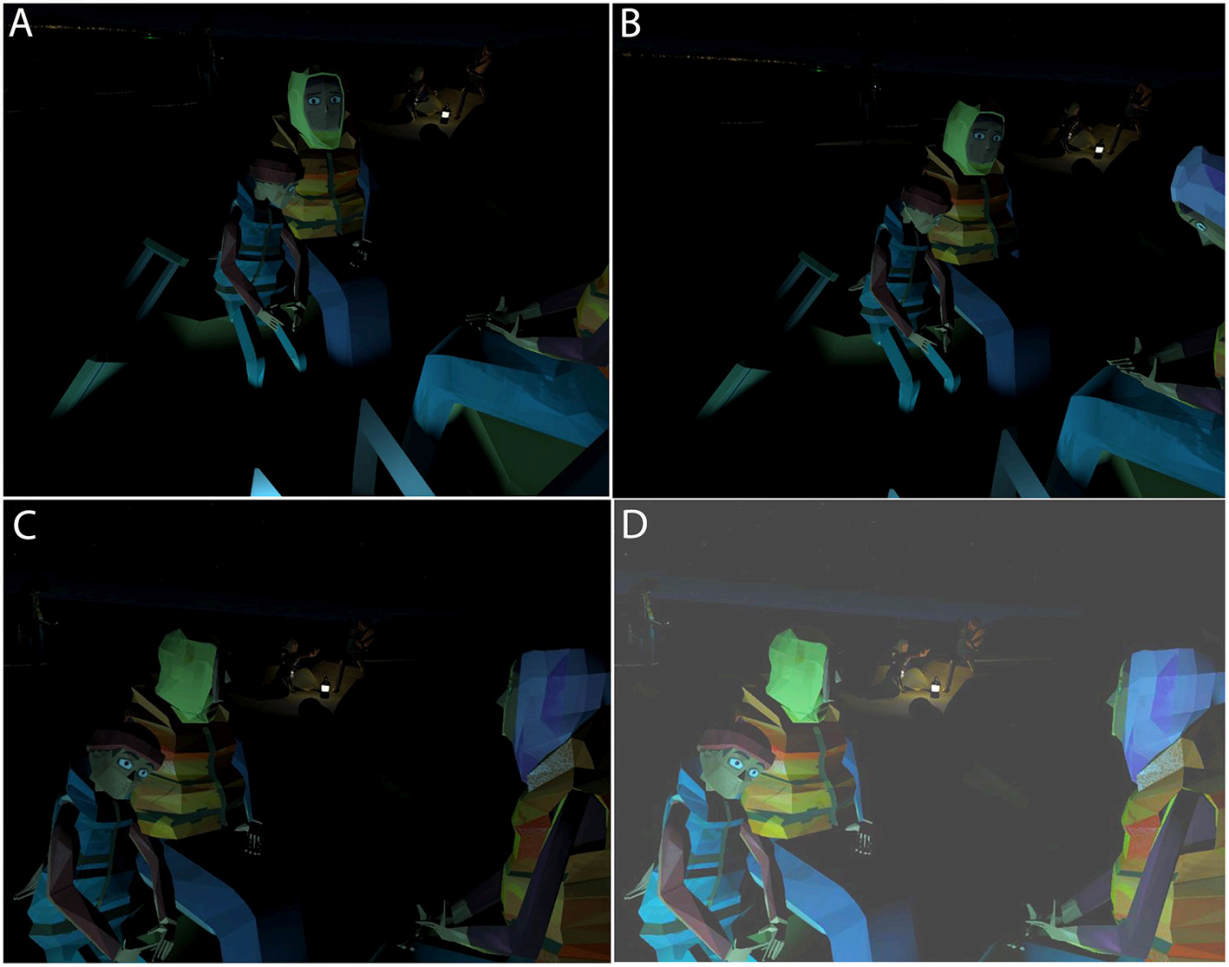
Partial illustration of the scenario. **(A)** The Responsiveness: *Look at* condition where the characters talk to the participant. **(B)** The Responsiveness: *None* condition where the character is not talking to the participant. **(C)** Other virtual characters in the scene. **(D)** The same as **(C)** but brightened for improved visualization.

Photorealism was an objective that was unattainable, so the creators chose a low polygon style of animation, with strong art direction to evoke the scenes depicted. The characters in the story were designed to have expressive eyes to strongly convey human emotion in response to gaze interaction (see Figure 1). Motion capture was used to ensure that character movement was as close to realistic as possible. When viewed on a flat screen the characters look like simplistic cartoons. However, in various demonstrations and public exhibitions, these avatars generated an empathic response. This was anecdotal observation though, and part of the purpose of the study was indeed to examine the effectiveness of the scenario.

Although the low polygon count animation reduced the need for detail, the 3D modeling closely referenced photographic and video footage of the migrant crisis. This included details such as the color of the life jackets. BBC reporters had highlighted the use of cell phones by migrants for updates on the best routes though Europe and to stay in touch with relatives. In “We Wait,” which is set at night, the cell phones are also used artistically to light faces.

Given the expense involved in creating a full CGI VR journalism experience such as “We Wait”, there was industry interest in understanding the audience benefit of techniques such as embodiment and responsiveness. Including a virtual body to represent the self in “We Wait” added to the production time—but the question is whether it added sufficient value to the experience to make this worth-while. Or should more production time have been allocated to creating higher fidelity graphics? Such questions justified interest in this study.

Another issue was the impact of self-representation on the participants' reactions to the scenes. Some virtual reality experiences are highly interactive and include some representation of the body. In many virtual reality experiences the participant is not embodied at all, but rather is a passive observer (e.g., in filmed content). In a journalistic setting, one constraint may be that the scene is largely narrative-based and thus non-interactive. It is thus not clear what the role of a self-representation would be in a non-interactive setting. In particular, it is not clear whether the participant can experience ownership over a body over which they have little control. This was a further motivation for the study.

### Presence

The illusion of being there (PI) and the illusion that the depicted events are really occurring (Psi) have been studied since the early 1990s. Factors originally postulated for the sense of “being there” include a wide field-of-view head-tracked stereoscopic experience, delivered at high framerate, with low latency, where actions of participants are reflected in changes to the virtual environment, and deployment of multiple sensory systems (visual, auditory, haptics; Heeter, 1992; Held and Durlach, 1992; Loomis, 1992; Sheridan, 1992, 1996; Steuer, 1992; Zeltzer, 1992; Barfield and Hendrix, 1995; Ellis, 1996; Slater and Wilbur, 1997). The fundamental purpose of this is so that *participants are able to perceive in the virtual environment via natural sensorimotor contingencies*. Sensorimotor contingencies (O'regan and Noë, 2001a,b; Noë, 2004) refer to the rules that we unconsciously employ in order to perceive: turn our head, bend down, look around, look over, reach out, touch, and so on. In this active theory of perception there is no perception without action—we construct our perception of reality through active movements and active engagement with the environment. To the extent that this is possible in a VR system so will the illusion of “being in” the place depicted by the virtual environment be likely to be engendered. Numerous studies have examined the technical components that a VR system must have to lead to this place illusion, summarized in a recent meta-study (Cummings and Bailenson, 2016).

Plausibility, the illusion that events are actually happening, is logically separable from Place Illusion: you can have the illusion of being in a place, but not accept the reality of events that are purportedly happening there. This happens, for example, when the behavior of virtual human characters are not believable since they do not respond to actions of the participant and are therefore ignored (Slater et al., 2006; Garau et al., 2008). Corroborating evidence is that observing at close hand a virtual musical string quartet, when the virtual performers looked toward the participant the level of plausibility was enhanced. Also the environmental auditory information matching visual elements in the scene also enhanced Psi (Bergstrom et al., 2017). The general issue of coherence between different aspects of the environment was also studied by (Skarbez et al., 2017). In their experiment participants had a virtual body, and the appropriateness of the body actions that could be carried out by the participant with respect to the surrounding events was the greatest contributor to Psi. On the same lines (Slater et al., 2010a) found that having a virtual body representation was an important contributor to both PI and Psi. Indeed the contribution of the virtual body representation to presence was one of the earliest potentially contributing factors studied (Slater and Usoh, 1994).

The third illusion, virtual body ownership, was first informally studied in the late 1980s by Jaron Lanier, and termed “homuncular flexibility”—see (Lanier, 2006; Won et al., 2015; Gonzalez-Franco and Lanier, 2017). This referred to the apparent flexibility of the brain's body representation to quickly adapt to new forms of body. In VR the participant's body can be replaced by a life-sized virtual one, which can appear to be human to more or less extent. The brain has a remarkable ability to take on extra-human or non-human attributes such as a tail (Steptoe et al., 2013), asymmetry (Kilteni et al., 2012), or more than two arms (Guterstam et al., 2011; Won et al., 2015).

The rubber hand illusion (Botvinick and Cohen, 1998) showed that a rubber hand can be felt as if it were a person's actual hand. This works though synchronization between seeing something repeatedly tap the visible rubber hand while feeling the tapping on the corresponding, but hidden, real hand. Temporal and spatial synchrony between the seen and felt tapping leads to a shift in proprioception from the real to the rubber hand, suggesting that the brain's body representation is partially based on moment to moment sensory contingencies (Armel and Ramachandran, 2003). Slater et al. (2008) showed that this illusion also works well in immersive virtual reality, and Sanchez-Vives et al. (2010) showed that synchrony between real and virtual hand movements is also effective. Yuan and Steed (2010) showed that having an interactive game that exercised visual-proprioceptive match led to the illusion of ownership over a virtual arm. This methodology of multisensory integration for body ownership can be extended to illusory ownership over the whole virtual body (Petkova and Ehrsson, 2008). Evidence suggests that the 1PP over the virtual body is particularly important (Slater et al., 2010b; Petkova et al., 2011), and that synchrony between real and virtual body movements is at least as effective in producing the illusion of body ownership as visual-tactile synchrony (Kokkinara and Slater, 2014).

The multisensory integration that gives rise to body ownership can also result in agency, that is the attribution of actions of the rubber arm or virtual body to the self—or the possibility that self-actions would lead to visible responses of the virtual body. For example, in the context of the rubber hand illusion Kalckert and Ehrsson (2012) show that although agency and body ownership can be dissociated, nevertheless seeing the rubber hand move in response to actual hand movements leads to agency over the rubber hand. Banakou and Slater (2014) showed that embodiment in a full virtual body through 1PP and visual-motor synchrony through real-time motion capture leads to body ownership and agency over the virtual body. Moreover, when the body spontaneously speaks (without the participants speaking) then there is agency over the speaking and an influence on how the participants actually speak after the VR exposure. More recent results have shown that when body ownership is induced through 1PP and visual-tactile synchrony, then even though there is no body movement (neither of the participant nor of the virtual body) participants nevertheless have the illusion of agency over the virtual body, but this is not reflected in any change in their subsequent speaking behavior (Banakou and Slater, 2017). Hence simply having body ownership over a virtual body seems to be associated with the illusion of agency over that body.

Overall there is evidence that the virtual body leads to greater presence. This was shown in an early study (Slater and Usoh, 1993), and it has been found that the virtual body contributes to both the Place Illusion and Plausibility aspects of presence (Slater et al., 2010a). More recently Steed et al. (2016) ran a study in the “wild” with an application for Google Cardboard and Gear VR devices. They found that having a virtual body increased some self-reports of presence, even though the virtual body was uncoordinated with any participant movements.

## Materials and methods

### Experimental design

Thirty two participants were recruited for the experiment and each was arbitrarily assigned to one of the 4 conditions Responsiveness × Embodiment, with 8 in each cell of the factorial table in a between groups design. There were 12 male participants out of the 32. The study was approved by the UCL Research Ethics Committee, and participants were required to give written informed consent.

### Materials

The virtual reality system was built on a Windows 8.1 computer with an Intel Core i7 processor, 8GB ram and a GeForce TitanX graphics card. The head-mounted display was an Oculus Rift CV1. No hand trackers or other hand-held devices were required for the experiment.

The virtual environment was originally built by Aardman Interactive using Unity 5.3.4p6 and written in C#. All modifications for the purpose of the experiment were done in the same version of Unity. All scenes were rendered at 90 Hz.

### Scenario

The “We Wait” experience consists of seven scenes, all set at night. In the first scene, the participant experiences an environment where they can see a distant horizon with lights and silhouettes of nearby people in the dark. There is background audio indicating a beach scene and some talking. Some short text captions appear in front of the participant to set the scene. The participant is told that the family to be shown in the next scene is fleeing from Syria, and they are waiting on a beach in Turkey for a boat to take them to Greece. In the second scene, a number of small lights come on (lamps, torches and mobile phone screens), and the participant can see that he or she is sitting on rocks facing a mother and child. There are various other adults seated or standing nearby. The mother starts to relate their story. In the third scene, the participant and the family are on a boat crossing the sea, but eventually the boat must turn back. In the fourth scene, the family is again on the beach waiting for another boat. In the fifth scene, they are again on a boat and this boat is caught by Turkish coastguards. In the sixth scene, the family is back on the beach again where some of the adults are discussing whether to wait for the boat again, which does finally appear. In the seventh scene, the characters disappear and text captions with some final statements and credits appear.

For this experiment, the participants experienced the full story. The only modifications were to vary elements to form conditions for the experiment. The first modification was to make the Responsiveness conditions. The *Look at* version is as originally designed. In particular, in the second and fourth scenes, the child occasionally buries his head in his mother's chest if the participant looks at him. Also in these two scenes, the mother appears to talk directly to the participant. In scenes two through five, some of the other adults will occasionally look back at the participant if she or he looks at them. In the Responsiveness: *None* version, none of the characters' behaviors are dependent on the participant and they never look toward the participant (except by chance). Additionally, in scenes two and four, the mother character is turned 15 degrees to her left so as to appear as if she is talking to another animated character, rather than the participant.

The original experience includes a simple virtual body self-representation of the participant seen from 1PP. The virtual body wears a life-jacket as do all the other characters in the piece. This was used for the Body condition. The virtual body representation animates based on head position only. When the head moves, the upper body is rotated so that the end of the neck is underneath the head position. The arms are shown resting on the knees and are not active. If the participant moves their head far enough that the upper body could not animate to follow, the body fades out. For the Non-Body condition there was no virtual body self-representation.

An outline of the scenario is available^6^ and readers can experience the Embodiment:*Body*, Responsiveness:*Look at* condition by downloading the “We Wait” app from the BBC's website^7^.

### Procedures

On arriving at the laboratory, participants were given an information sheet about the study. They were asked to read this and then read and sign a separate consent form. The experimenter then explained the equipment to be used and helped the participant put on the head-mounted display. Each participant was then asked to view the “We Wait” VR in one of four experimental conditions. Participants were assigned to their condition by order of arrival.

After the virtual environment experience the participant completed a questionnaire. Finally, the experimenter added a URL shortcut to the participant's phone. This shortcut would appear as an icon on the home screen of their smartphone, whether iOS or Android-based. This URL referred to a server at UCL that the authors controlled. The URL coded the participant ID as a parameter. This server performed a HTTP 302 redirect to a BBC news feed about the refugee crisis^8^. This redirect did not contain the participant ID. The server logged all redirects.

Upon completion of the experiment, the participants were paid £5 (approximately $7.5 US) for their participation. The experiment took about 15 min.

### Response variables

Prior to their virtual reality exposure participants completed a pre-experience questionnaire on demographic aspects (age, sex, previous VR experience, the extent of computer game playing, and the extent to which they followed current affairs).

At the end of their VR exposure participants were given a post-experience questionnaire, to elicit their responses on presence (PI and Psi), body ownership and agency (Table 1). Note that Psi is divided into two aspects—the plausibility of the situation, and plausibility of the virtual human characters.

**Table 1:** Post Experience Questionnaire.

***Variable name***	***Question***
**(A) PLACE ILLUSION**	
*There*	Please rate your sense of being in the boat and on the beach, on the following scale from 1 to 7, where 7 represents your normal experience of being in a place.
*Real*	To what extent were there times during the experience when the boat and on the beach was the reality for you?
*Visited*	When you think back about your experience, do you think of the boat and beach more as images that you saw, or more as somewhere that you visited?
lab^*^	During the time of the experience, which was strongest on the whole, your sense of being in the boat and beach, or of being in the real world of the laboratory?
*Overwhelm*	During the time of the experience, did you often think to yourself that you were just sitting in a laboratory or did the experience overwhelm you?
**(B) PLAUSIBILITY OF THE SITUATION**	
*Behavereal*	How much did you behave within the scene as if the situation were real?
*Emotionreal*	How much was your emotional response the same as if it had been real?
*Thoughtsreal*	How much were the thoughts you had within the experience the same as if it had been a real situation?
*Behaveasifreal*	How much were you thinking things like “I know this isn't real” but then surprisingly finding yourself behaving as if it was real?
*Physicalreal*	To what extent were your physical responses within the experience (e.g., heart rate, blushing, sweating, etc.) the same as if it had been a real situation?
*Experiencereal*	Overall how much did you treat the experience as if it were real?
**(C) PLAUSIBILITY OF THE VIRTUAL PEOPLE**	
*Peoplereal*	How much did you behave as if the virtual people were real?
*Emotionpeople*	How much was your emotional response to the virtual people as if they were real?
*Thoughtspeople*	How much were your thoughts in relation to the virtual people as if they were real?
*Physicalpeople*	How much did you have physical responses (such as change in heart rate, blushing, sweating, etc.) to the virtual people as if they were real?
*Behavingasifpeople*	How much were you thinking things like “I know these people are not real” but then surprisingly finding yourself behaving as if they were?
**(D) BODY OWNERSHIP AND AGENCY**	
*Mybody*	During the experience I felt that the body I saw when looking down toward myself was my own body (even though it didn't look like me).
Twobodies^*^	During the experience I felt as though I had two bodies.
*Agency*	During the experience I felt that the movements of the virtual body were my movements.
Otherbody^*^	During the experience I felt that the virtual body belonged to someone else.
**(E) INTEREST IN CURRENT AFFAIRS**	
*Affairs*	How interested are you in news and current affairs?

Each question was scored on a 1-7 Likert Scale, where 1 indicates least agreement and 7 most agreement with the proposition indicated by the question.

* indicates reverse interpretation, where higher values indicate less agreement with the item of measurement such as “Place Illusion.”

The questions regarding body ownership and agency have been used several times before - for example, (Banakou et al., 2013, 2016; Banakou and Slater, 2014, 2017; Bergström et al., 2016; Hasler et al., 2017; Tajadura-Jiménez et al., 2017). The motivation for the *agency* question was to see whether there was any illusory agency as was found in (Banakou and Slater, 2017) which used embodiment based on 1PP and visuotactile synchronous stimulation. In the current experiment there was no other stimulation than the 1PP view over the body, in the “Body” condition. The motivation for *twobodies* was that the illusion of having two bodies would be evidence against the type of body ownership that we were interested in generating—where participants would feel ownership only over their virtual body rather than both the virtual and real body. Body ownership has also been studied with respect to a third person perspective (3PP) view over a virtual body in the context of out-of-the-body illusions—for example, (Ehrsson, 2007; Lenggenhager et al., 2007; Bourdin et al., 2016), but this was not the goal here.

After the experiment participants were given access to the news feed containing further information about the refugee crisis using the method described above. They were invited to access the web page subsequent to the experiment. We recorded how many times after the first that they visited the web site (*webvisits*) and the time in *seconds* after the first (required) visit. There could be many reasons for multiple visits–to look up the information again, or because someone was interrupted the first time that they looked, or the participant had a subsequent thought about the issue and wanted to check something, or had forgotten the name of one of the books cited etc. The fundamental motivation for this measure was the idea was that the more visits the more the determination to find the information.

### Statistical methods

#### Factor analysis for the questionnaire responses

In order to reduce the number of questionnaire variables a factor analysis, with varimax rotation, was carried out on each set in the tables above. Box plots of all the raw questionnaire scores are available in Supplementary Information. The interpretation of each factor was identified where possible, and corresponding factor scores were used. This also has the advantage of transforming the ordinal variables into continuous ones. Normally it would not be advisable to carry out a factor analysis on ordinal variables, but as a test Polychoric PCA analysis was also used (this treats ordinal variables as if they were derived from cut-offs sampled from a Normally distributed variable; Olsson, 1979) and scores derived from those. In each case there was a very high correlation between the factor analysis scores and the Polychoric PCA scores. The factor analysis was carried out using Stata 15 software.

#### Overall method

Bayesian analysis was used where all responses are treated simultaneously in one overall model. (Classical statistics would use a separate model for each variable and hence lose all control over the “significance level”).

Suppose we have *p* response variables *y*_1_, …, *y*_*p*_. We have *n* observations on each variable, so that e.g., *y*_*mi*_, *i* = 1, …, *n* are the observations on response variable *y*_*m*_. Suppose that the independent/explanatory variables are *x*_1_, …, *x*_*k*_ (not all explanatory variables need be used for each response variable). Then the model is of the form;

$$\begin{array}{l} y_{1i}\sim N\left(\mu_{1i},\sigma_{1}\right)\\ y_{2i}\sim N\left(\mu_{2i},\sigma_{2}\right)\\ \;\ldots \\ y_{pi}\sim N\left(\mu_{pi},\sigma_{p}\right) \end{array}$$

where

(1) $$\begin{array}{l} \mu_{ri}=\;\beta_{r0}+\;\beta_{r1}x_{i1}+\ldots +\beta_{rk}x_{ik}\\ \;i=1,\ldots ,n,r=1,2,\ldots ,p \end{array}$$

*N*(μ, σ) refers to the Normal distribution with mean μ and standard deviation σ. The unknown parameters β and the standard deviations are given a wide prior distribution. The Bayesian method will use the data to update the distributions of the parameters from which we can make inferences. For example, suppose that the posterior probability *P*(β_13_ > 0 |*Data*) is high (e.g., greater than 0.9, but ultimately the interpretation is up to the reader) then this is evidence that the variable *x*_3_ positively influences *y*_1_.

The model was run using the Stan software (Carpenter et al., 2017) through the Matlab interface^9^. All models were run using 2000 iterations and 4 chains.

## Results

### Factor analysis of the questionnaires

Here we show how the factor analysis was used on the questionnaires. Three factors were retained in the case of the Place Illusion questions from Table 1, and the factor loadings on the three scoring variables (*ypi1*-*ypi3*) are shown in Table 2.

**Table 2:** Factor Analysis for Place Illusion, resulting in three retained factors *F1, F2, F3, and the corresponding scoring coefficients for the factor scores yp1, yp2, and yp3*.

	**Factor loadings**			**Scoring coefficients**		
**Variable**	***F*1**	***F*2**	***F*3**	***ypi*1**	***ypi*2**	***ypi*3**
*there*	0.72	0.02	0.01	0.38	−0.20	−0.13
*real*	0.76	0.33	0.16	0.52	0.17	0.10
*visited*	0.30	0.57	0.23	−0.03	0.41	0.17
*lab*	−0.19	−0.50	0.14	0.01	−0.31	0.21
*overwhelm*	0.30	0.13	0.43	0.04	−0.02	0.36
Interpretation: The illusion of …	“… being there in the real place.”	“… visiting the boat and beach, not being the lab.”	“… being overwhelmed by the experience”.			

The factor loadings can be thought of as how much the factor “explains” the variance in the original variables, and the scoring coefficients are the coefficients of the equations that describe the factor scores in terms the linear combination of the original variables. The first factor (*F1*) is dominated by *there* and *real*, which is also reflected in the corresponding scoring coefficients for *ypi1* and we can interpret this factor as representing “being there in a real place.” The second factor has comparatively lower factor loadings, but can be interpreted as “visiting the boat and beach, not being in the lab” (recalling that *lab* is reverse coded with respect to Place Illusion). F3 has no obvious interpretation other than the experience being overwhelming, and the factor loadings are all low.

Table 3 shows the results for the plausibility of the situation. *F1* is dominated by *behavereal* and *thoughtsreal* representing “Real behavior and thoughts about the situation.” *F2* refers to being surprised at behaving realistically, with corresponding physical responses and experiencing the scenario as real, but not actually behaving realistically or having corresponding emotions and thoughts. This is consistent with the dominant variable which is experiencing the situation as real. *F3* is dominated by the emotion variable.

**Table 3:** Factor Analysis for Plausibility of the Situation resulting in three retained factors and corresponding scoring coefficients *ypsi_sit1-ypsi_sit3*.

	**Factor loadings**			**Scoring coefficients**		
**Variable**	***F*1**	***F*2**	***F*3**	***ypsi_sit*1**	***ypsi_sit*2**	***ypsi_sit*3**
*behavereal*	0.74	0.31	0.09	0.55	−0.18	−0.30
*emotionreal*	0.23	0.22	0.57	−0.05	−0.11	0.40
*thoughtsreal*	0.62	0.24	0.33	0.27	−0.14	0.20
*behaveasifreal*	0.30	0.41	0.02	0.03	0.13	−0.11
*physicalreal*	0.24	0.66	0.08	−0.11	0.34	−0.19
*experiencereal*	0.54	0.65	0.37	0.09	0.64	0.41
Interpretation	“Real behavior and thoughts about the situation.”	“Experiencing the situation as if real with corresponding physical responses.”	“Experiencing the situation with emotions as if real.”			–

In Table 4 the factor F1 is dominated by *physicalpeople* and *behavingasifpeople*–responding physiologically and behaviourally as if the people were real. The factor *F2* is dominated by the people taken as real with corresponding emotional and thinking responses, but without the behavioral response. So this can be interpreted as “Thoughts and emotions as if the people were real.”

**Table 4:** Factor analysis of the Plausibility of the virtual people resulting in two retained factors and corresponding scoring coefficients *ypsi_people1 and ypsi_people2*.

	**Factor loadings**		**Scoring coefficients**	
**Variable**	***F*1**	***F*2**	***ypsi_people*1**	***ypsi_people*2**
*peoplereal*	0.45	0.53	0.03	0.25
*emotionpeople*	0.42	0.54	0.01	0.26
*thoughtspeople*	0.38	0.62	−0.08	0.39
*physicalpeople*	0.74	0.44	0.48	0.07
*behavingasifpeople*	0.74	0.23	0.41	−0.19
Interpretation	“Responding physically and behaviourally as if the people were real.”	“Thoughts and emotions as if the people were real.”		

The results for body ownership are shown in Table 5. These factors make no sense (compared to all previous experiments). “*twobodies*” and “*otherbody*” should be negative (since these are reversed scored questions). Not shown here but the correlation between *mybody* and each of *twobodies* and *otherbody* are both positive (they should be negative). Hence we conclude that no measurable notion of “body ownership” arose (see also the graphs below) and this is not analyzed further.

**Table 5:** Body Ownership and Agency resulting in scoring coefficients *yownership1, yownership2*.

	**Factor loadings**		**Scoring coefficients**	
**Variable**	***F*1**	***F*2**	***yownership*1**	***yownership*2**
*mybody*	0.57	0.26	0.34	0.19
*twobodies*	0.51	0.22	0.27	0.14
*agency*	0.06	0.51	−0.01	0.40
*otherbody*	0.57	−0.19	0.37	−0.24

### Graphical results

**Figure 2:**
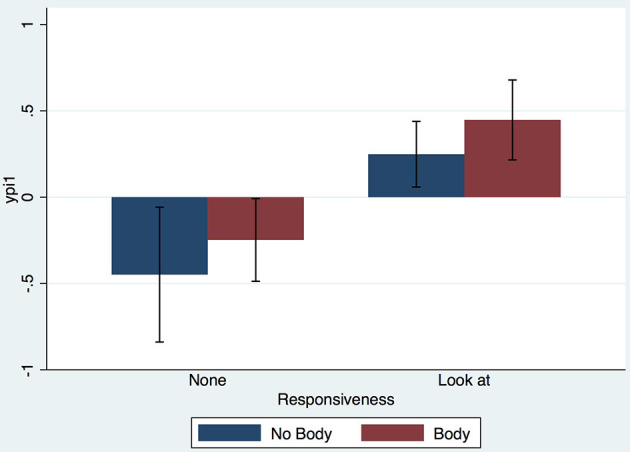
Bar chart of the *yp1* factor score, showing means and standard errors.

**Figure 3:**
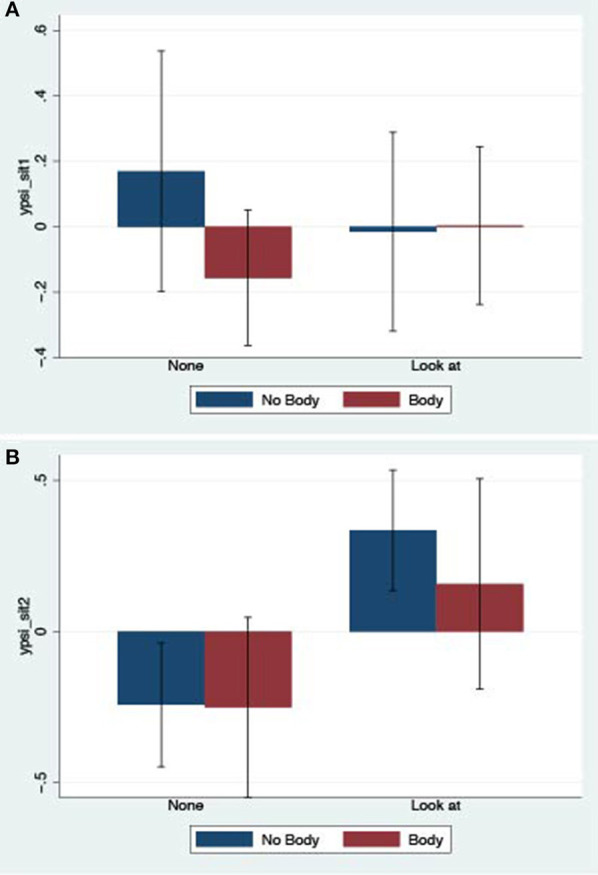
Bar charts of means and standard errors for the factor scores for Plausibility of the situation. **(A)** *ypsi_sit1*: real behavior and thoughts about the situation. **(B)** *ypsi_sit2*: being surprised at behaving realistically.

**Figure 4:**
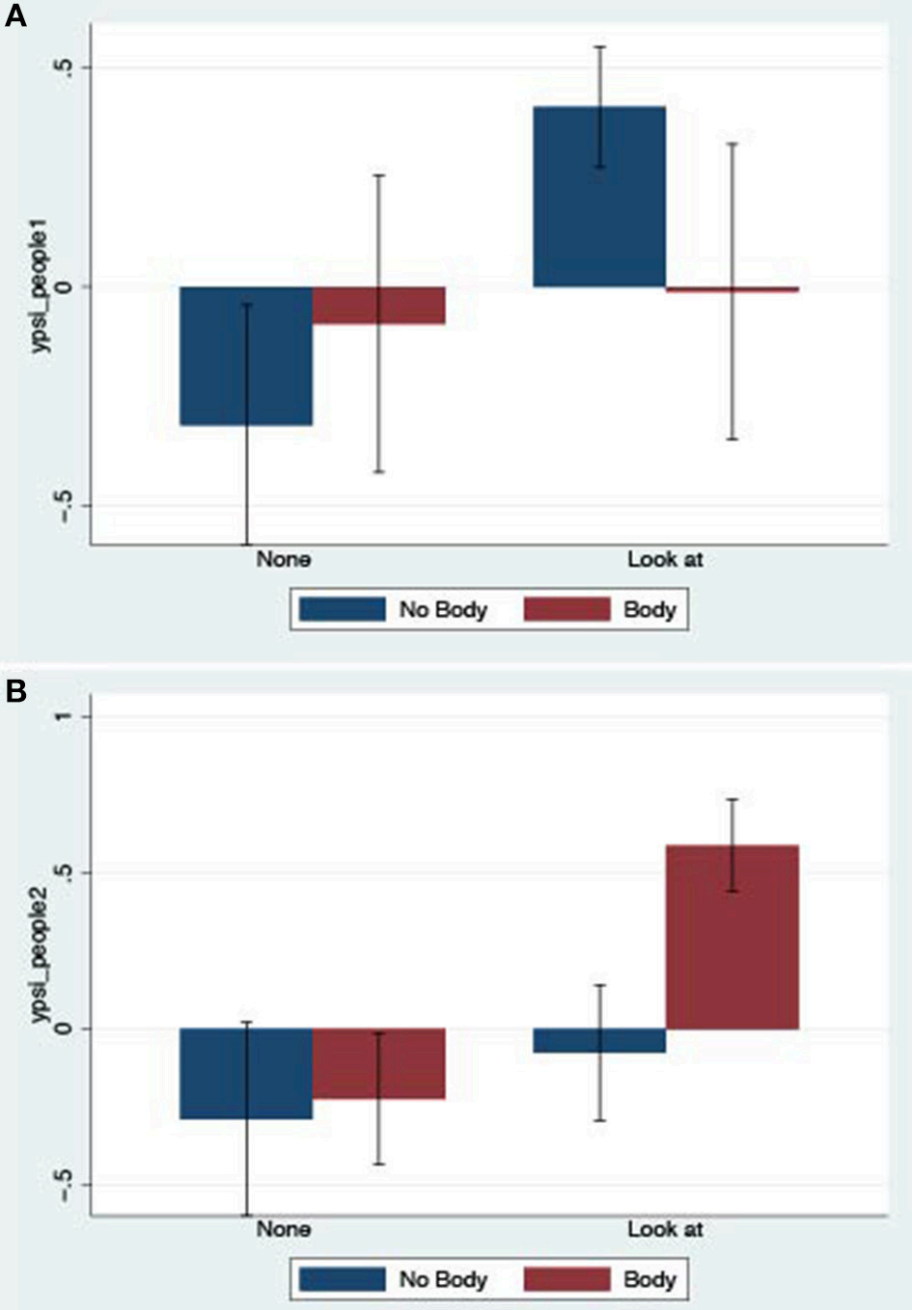
Bar charts of means and standard errors for Plausibility of the virtual people. **(A)** *ypsi_people1*—responding physically and behaviourally as if the people were real. **(B)** *ypsi_people2*-thoughts and emotions as if the people were real.

Here we show the means and standard errors of the derived factor analysis variables. The formal analysis of these is given in the next section. Figure 2 shows the bar chart of the means and standard errors of the derived *ypi1* factor. Clearly there is no effect of Embodiment but there is an effect of Responsiveness. The bar chart for *ypsi_sit1* (Figure 3A) shows no effect of the factors, since the variances (illustrated by the standard error bars) are so high. However, *ypsi_sit2* shows a likely effect of Responsiveness (Figure 3B). The bar chart for *ypsi_people1* (Figure 4A) shows a strong effect of Responsiveness, but it is less clear for Embodiment. The bar chart for *ypsi_people2* (Figure 4B) shows a strong effect of Embodiment, but not of Responsiveness.

### Web visits

Eight out of the 32 participants visited the web again after the initial (required) visit. A question relevant to this asked in the pre-exposure questionnaire (on a scale of 1–7) is the *affairs* question shown in Table 1E.

**Figure 5:**
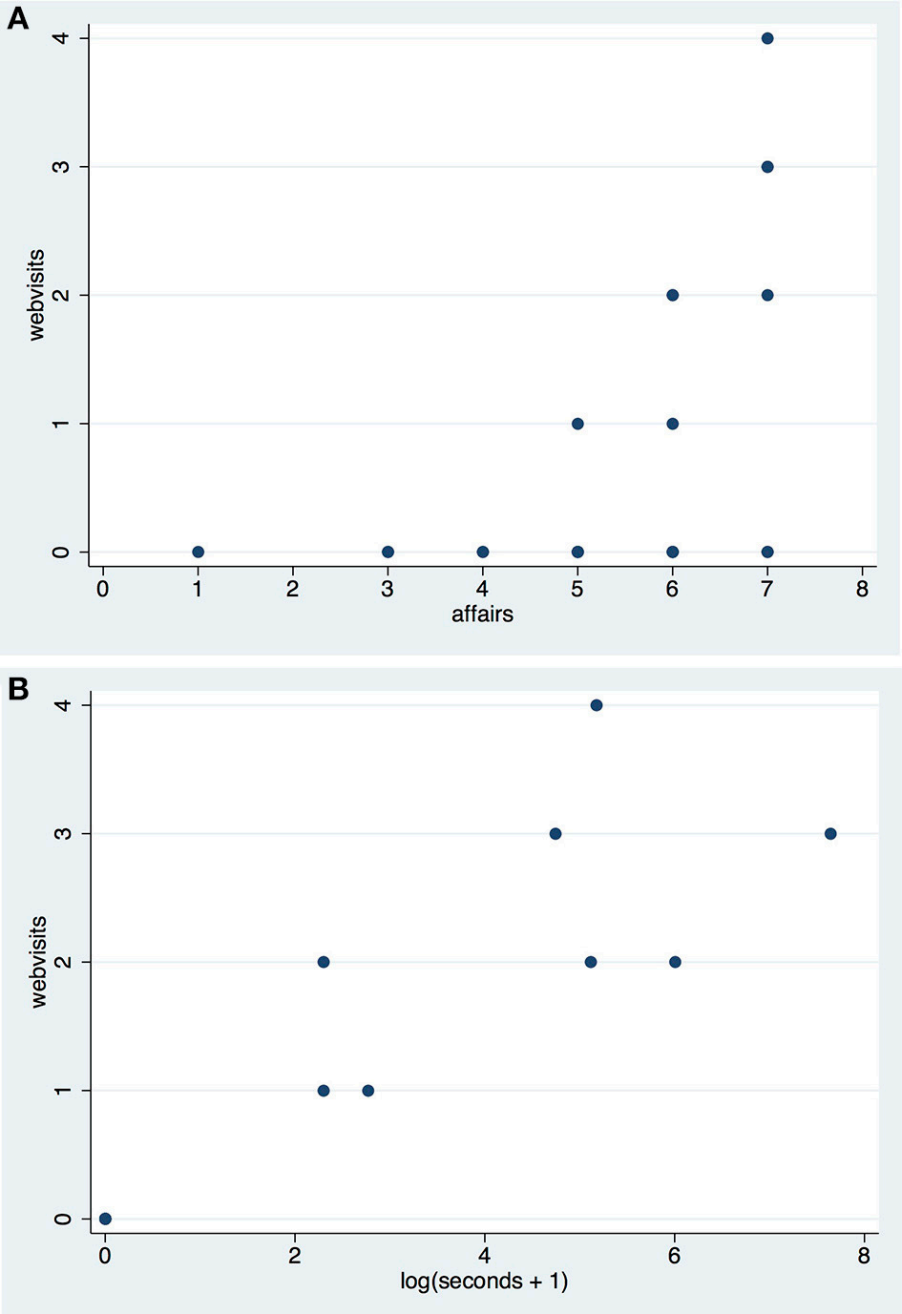
Scatter diagram of the number of *webvisits* **(A)** by *affairs* **(B)** log(*seconds*+1).

All of the 12 males gave scores of 5 or more on this question, compared with 14/20 females. For those who did visit the web there is the possibility of a relationship with *affairs*—the greater interest in current affairs the more likely to visit the web page. This is illustrated in Figure 5A. Moreover 6/12 males had 0 web visits whereas 18/20 females had 0 web visits. Both interest in current affairs and the sex of participants seem to play a role in *webvisits*, and these need to be taken into account in the analysis. For the response variable *seconds* we use log(*seconds*+1) since on this log scale there is a linear relationship with web visits (Figure 5B). Note that when there are zero visits then *seconds* = 0, and log(*seconds*+1) = 0.

### Model results

Table 6 gives the results for the complete model. Each block represents a specification of the form as in 3.2 above. For example,

$$\begin{array}{l} y_{pi1,i}\sim N(\beta_{ypi1,0\;}+\;\beta_{ypi1,1\;}Responsiveness_{i}\\ \;+\;\beta_{ypi1,2\;}Embodiment_{i},\;\sigma_{ypi}) \end{array}$$

where

(2) $$\begin{array}{l} \text{Responsiveness is coded by }\left(\text{None}=0,\text{Lookat}=1\right),\text{ and}\\ \text{ Embodiment by }\left(\text{No Body}=0,\text{Body}=1\right) \end{array}$$

Note that a common standard deviation has been used within each block, so that the results for those are put at the end of each block. (Allowing different standard deviations for each section does not change the results). Similarly for the other response variables except for *webvisits* and *lseconds* = log(*seconds*+1).

**Table 6:** Means, Standard Errors and 95% credible intervals for the parameters of Equation 1.

**Parameter (coefficient of)**	**Mean**	**S.E. mean**	**2.5%**	**97.5%**	Prob*
**ypi1**					
β_*ypi*1, 0_	−0.45	0.0032	−0.84	−0.05	0.011
β_*ypi*1, 1_(Responsiveness, None = 0, Lookat = 1)	0.69	0.0037	0.22	1.15	0.999
β_*ypi*1, 2_(Embodiment: No Body = 0, Body = 1)	0.20	0.0037	−0.28	0.67	0.803
**ypi2**					
β_*ypi*2, 0_	0.07	0.0031	−0.32	0.47	0.643
β_*ypi*2, 1_(Responsiveness)	−0.15	0.0036	−0.60	0.29	0.256
β_*ypi*2, 2_(Embodiment)	0.00	0.0037	−0.46	0.45	0.510
**ypi3**					
β_*ypi*3, 0_	−0.13	0.0032	−0.52	0.26	0.247
β_*ypi*3, 1_(Responsiveness)	0.21	0.0037	−0.24	0.68	0.817
β_*ypi*3, 2_(Embodiment)	0.06	0.0037	−0.39	0.53	0.599
σ_*ypi*_	0.66	0.0008	0.57	0.77	
**ypsi_sit1**					
β_*ypsi*_*sit*1, 0_	0.08	0.0036	−0.35	0.52	0.649
β_*ypsi*_*sit*1, 1_(Responsiveness)	−0.01	0.0042	−0.54	0.50	0.484
β_*ypsi*_*sit*1, 2_(Embodiment)	−0.15	0.0042	−0.67	0.35	0.284
**ypsi_sit2**					
β_*ypsi*_*sit*2, 0_	−0.21	0.0036	−0.66	0.24	0.183
β_*ypsi*_*sit*2, 1_(Responsiveness)	0.49	0.0042	−0.03	1.00	0.969
β_*ypsi*_*sit*2, 2_(Embodiment)	−0.09	0.0041	−0.61	0.43	0.368
**ypsi_sit3**					
β_*ypsi*_*sit*3, 0_	−0.03	0.0036	−0.48	0.42	0.440
β_*ypsi*_*sit*3, 1_(Responsiveness)	0.07	0.0042	−0.45	0.60	0.616
β_*ypsi*_*sit*3, 2_(Embodiment)	0.00	0.0040	−0.50	0.49	0.494
σ_*ypsi*_*sit*_	0.74	0.0009	0.64	0.87	
**ypsi_people1**					
β_*ypsi*_*people*1, 0_	−0.15	0.0036	−0.60	0.29	0.238
β_*ypsi*_*people*1, 1_(Responsiveness)	0.40	0.0041	−0.11	0.92	0.939
β_*ypsi*_*people*1, 2_(Embodiment)	−0.09	0.0040	−0.59	0.40	0.360
**ypsi_people2**					
β_*ypsi*_*people*2, 0_	−0.44	0.0036	−0.89	0.02	0.030
β_*ypsi*_*people*2, 1_(Responsiveness)	0.51	0.0042	−0.01	1.04	0.973
β_*ypsi*_*people*2, 2_(Embodiment)	0.36	0.0040	−0.14	0.87	0.924
σ_*ypsi*_*people*_	0.74	0.0011	0.62	0.89	
**WEBVISITS**					
β_*webvisits*, 0_	−3.10	0.0644	−11.14	4.92	0.222
β_*webvisits*, 1_sex (M = 0, F = 1)	−0.44	0.0222	−3.45	2.02	0.404
β_*webvisits*, 2_affairs	0.59	0.0092	−0.56	1.71	0.847
β_*webvisits*, 3_(Responsiveness)	−0.17	0.0102	−1.45	1.10	0.397
β_*webvisits*, 4_(Embodiment)	−0.44	0.0121	−2.00	1.00	0.283
σ_*webvisits*_
θ	0.73	0.0012	0.58	0.87	
**log(seconds**+**1)**					
β_*lseconds*, 0_	−5.66	0.0462	−11.29	0.16	0.027
β_*lseconds*, 1_affairs	1.29	0.0073	0.36	2.17	0.998
β_*lseconds*, 2_sex	4.66	0.0469	−1.34	10.31	0.937
β_*lseconds*, 3_affairs × sex	−1.10	0.0078	−2.03	−0.10	0.017
β_*lseconds*, 4_ Embodiment	0.65	0.0097	−0.58	1.85	0.866
σ_*lseconds*_	1.70	0.0039	1.29	2.27	
					

Prob is the posterior probability of the corresponding parameter being positive, i.e., P(β_*_ | Data) > 0.

For each of the β parameters the prior distribution is N(0,5), giving a range of possibilities from approximately −15 to 15. For the σ parameters a half-Cauchy distribution is used [i.e., Cauchy but restricted to the interval (0, ∞)].

The variable *webvisits* is in a special category. It is a count variable and would normally be modeled by a Poisson distribution. However, there are a large number of 0 responses. This is called “zero inflated Poisson regression.” The model is as follows:

*webvisits* is 0 with probability θ, and for values 1 or more has a Poisson distribution, truncated at lower bound 1, with probability 1–θ. The Bayesian method can estimate both θ and the mean of the Poisson distribution which is set equal to the linear model. Note, therefore, that θ is the probability of 0 web visits. The prior distribution for θ was chosen to be uniform over the range [0,1].

For *webvisits* and for *lseconds* = log(*seconds*+1) we introduce both “sex” and “affairs” as explanatory variables, given the discussion at the end of Section Web visits. Note that all prior probabilities (corresponding to the right-most column of posterior probabilities in Table 5) are 0.5, and all prior 95% credible intervals for the β parameters are −9.8 to 9.8.

### Interpretation of results

*ypi1* (interpreted as the illusion of being there in a real place) is positively associated with Responsiveness (Prob = 0.999).

*ypsi_sit2* (experiencing the situation as real) is positively associated with Responsiveness (Prob = 0.969).

*ypsi_people1* (responding physiologically and behaviourally as if the people were real) is positively associated with Responsiveness (Prob = 0.939).

*ypsi_people2* (thoughts and emotions as if the people were real) is positively associated with both Responsiveness and Embodiment (Prob = 0.973 and 0.924 respectively).

Note that the mean estimate of θ is 0.73, which corresponds well with the observed data (0.75).

*lseconds* (log time between first and last web site access) is positively associated with *affairs* (how the news is followed) (prob = 0.998), but the amount of news following when female is negatively associated with the last time to web access, shown by the interaction term (prob = 1–0.017 = 0.983).

Some less well supported possibilities are:

Having the body is positively associated with *ypi1* (prob = 0.803) and positively associated with *lseconds* (prob = 0.866).

*ypi3* (interpreted as being overwhelmed by the experience) may be positively associated with responsiveness (prob = 0.817).

### Composite results

An advantage of the Bayesian method is that we can derive probabilities of further statements from the joint distribution of all the parameters. Of particular interest is the probability that Responsiveness positively affected at least one of the questionnaire responses and also the probability that Embodiment positively affected at least one of the questionnaire responses. For example, the first is the probability

$$\begin{array}{l} P((\beta_{ypi1,1\;}>0)\;\vee \;(\beta_{ypi2,1\;}>0)\;\vee \;\cdots \\ \vee \;(\beta_{ypsi_{people2,1}}>0)|\;Data) \end{array}$$

(where ∨ means “or”) i.e., the probability that at least one of the coefficients of PI or Psi is positive. Some pertinent probabilities are given in Table 7. For example, it is almost certain (probability 1.000) that Responsiveness positively influenced at least one of the presence outcomes. It can be seen that the factors did influence almost all the outcomes, except for Embodiment which had a lower probability of influencing Psi situation.

**Table 7:** Posterior Probabilities of the factors (Responsiveness, Embodiment) influencing various outcomes.

**Expression**	**Probability for Responsiveness**	**Probability for Embodiment**
The factor contributes positively to at least one of the presence (PI and PSI) questions.	1.000	1.000
The factor contributes positively to at least one of the PI responses.	1.000	0.962
The factor contributes positively to at least one of the PSI Situation responses.	0.995	0.757
The factor contributes positively to at least one of the PSI People questions.	0.997	0.944

## Discussion

In this study we have examined the effect of responses of virtual characters in the scenario to the participant, and also self-representation of the participant with a virtual body on Place Illusion, Plausibility, body ownership, and the extent of follow-up of news items about the refugee crisis. Overall the levels of presence (Place Illusion and Plausibility) were high (comparable with past findings). The assessment of body ownership (Table 1D) yielded inconsistent results and was not used. In what follows we refer to a 0.9 probability threshold as “strong,” and between 0.8 and <0.9 as “moderate.” We found that the Responsiveness: *Look at* factor (where characters looked at the participant) positively and strongly influenced the illusion of being in a real place, experiencing the situation as real, responding physiologically and behaviourally as if the people were real and having thoughts and emotions as if the people were real. The virtual body self-representation moderately influenced the illusion of being in a real place, and the time to the last web page visit. Overall Responsiveness was the dominant factor and contributed positively to the key responses. The Virtual Body contributed far less. However, there is strong evidence that both factors contributed positively to both Place Illusion and Plausibility.

Regarding the number of web visits and the time to the last visit, only 25% of participants did so. However, the Director of Audiences for the BBC, Mr Nick North, was surprised by these results writing to the authors: “*We know how difficult it can be to drive audiences to online content; from all the BBC's research to date we might expect a small proportion of the audience to go online to a related website after watching a TV programme, for example. So, whilst this was a small study, a 25% conversion rate from the We Wait VR experience is very impressive, and potentially indicative of the significant impact VR could have at scale.”*

There was also clear evidence that the sex of participants influenced their follow-up (men more likely to do so than women) and that general interest in current affairs also was important (with men in this sample showing more interest overall in current affairs). The only factor that had some moderate influence on this was Embodiment.

It is noteworthy that the illusion of being there in the real place (*ypi1*) was strongly influenced by the Responsiveness factor, and having thoughts and emotions as if the virtual characters were real (*ypsi_people2*) was strongly positively influenced by both factors. These were the most compelling results.

The overall recommendation from this study is that if the scenario permits then making characters respond to actions of the participants (such as looking back at them when the participants look at the characters) is highly beneficial, and there is some evidence that having the virtual body can be beneficial for presence and possibly for the follow-up action. One purpose of these types of scenario is for education purposes, to encourage people to find out more. The virtual body may contribute to this after eliminating the major effects of sex and interest in current affairs. Since, as we have seen, the virtual body contributes to the feeling that the virtual characters were real, and also contributes to Place Illusion (*ypi1*) it makes sense that it would be likely to contribute to the follow-up action. It is as if there was the implicit inference by participants: *These were real people, I was amongst them—so I had better find out more about what is going on*.

The failure of the Virtual Body to elicit the illusion of body ownership is not surprising in relation to previous findings. In prior experiments there was always a period of explicit embodiment, where participants were asked to observe their virtual bodies by directly looking down toward them and in a virtual mirror, combined with either synchronous visuotactile stimulation (Slater et al., 2010b), or visuomotor stimulation (Yuan and Steed, 2010;Banakou et al., 2013), or both (Kokkinara and Slater, 2014). In the present experiment there was no embodiment period at all where people were expressly directed to observe their virtual body with some period of multisensory stimulation, such as moving while the virtual body moved synchronously. Nevertheless, as found long ago, simply having a virtual body may positively influence presence (Slater and Usoh, 1994).

We pointed out earlier that VR for documentary or news reporting inevitably is based on a narrative, where the participant may not intervene. Indeed intervention may be counter to news values, since it could be seen as changing the story itself rather than faithful reporting. Nevertheless, the results of our study suggest that some minimal changes to the scenario can be positive: make the characters involved in the scenario look toward the participant especially in response to participant actions, and give the participant a body. The first will contribute specifically to plausibility and the second overall to presence overall. Fostering the sense in the participant that they were “there” and what was happening was “real” might be helpful in engendering follow-up and further interest in the news story.

## Author contributions

MS, AS, and ZW conceived and designed the experiment. YP carried out the experiment and collected and compiled the data. AS and YP modified the implementation of the scenario for the experiment. MS carried out the analysis of the results. MS with the help of all authors wrote the paper. The manuscript was reviewed by all authors.

### Conflict of interest statement

The authors declare that the research was conducted in the absence of any commercial or financial relationships that could be construed as a potential conflict of interest. “We Wait” was originally created for artistic, demonstration and testing purposes, and this study was carried out subsequent to the original work. This study was not envisaged as part of the original work. There are no financial interests for the BBC regarding this study.

## Footnotes

^1^https://www.youtube.com/watch?v=_z8pSTMfGSo&t=18s

^2^https://www.youtube.com/watch?v=3YcyzS-R_Oc

^3^https://www.ted.com/talks/chris_milk_how_virtual_reality_can_create_the_ultimate_empathy_machine

^4^https://sheffdocfest.com/films/6103

^5^http://www.broadcastdigitalawards.co.uk/winners/ and https://www.broadcastnow.co.uk/home/broadcast-digital-awards/best-vr-experience-we-wait/5119627.article

^6^https://www.youtube.com/watch?v=3YcyzS-R_Oc

^7^http://www.bbc.co.uk/taster/projects/we-wait

^8^http://www.bbc.co.uk/news/topics/23672ac3-fcad-42c7-a557-23a954eb0e7b/europe-migrant-crisis

^9^http://mc-stan.org/users/interfaces/matlab-stan
