# Supplementary material for: “We Wait”—The Impact of Character Responsiveness and Self Embodiment on Presence and Interest in an Immersive News Experience
Source: Front Robot AI. 2018 Oct 4;5:112. doi: 10.3389/frobt.2018.00112 (PMC7805673; doi:10.3389/frobt.2018.00112)
Supplement: Supplementary file 1 [file Data_Sheet_1.pdf]

‘We Wait’ – The Impact of Character Responsiveness and Embodiment on Presence and Interest in an Immersive News Experience

Anthony Steed, Ye Pan, Zillah Watson, Mel Slater

Supplementary Material

Figure S1 shows the distribution of scores for Place Illusion. It can be seen that especially in the case of *there* and *real* the scores are quite high, and highest for Interact + Body in the case of *there*. *Lab* being a reverse coded response is low.

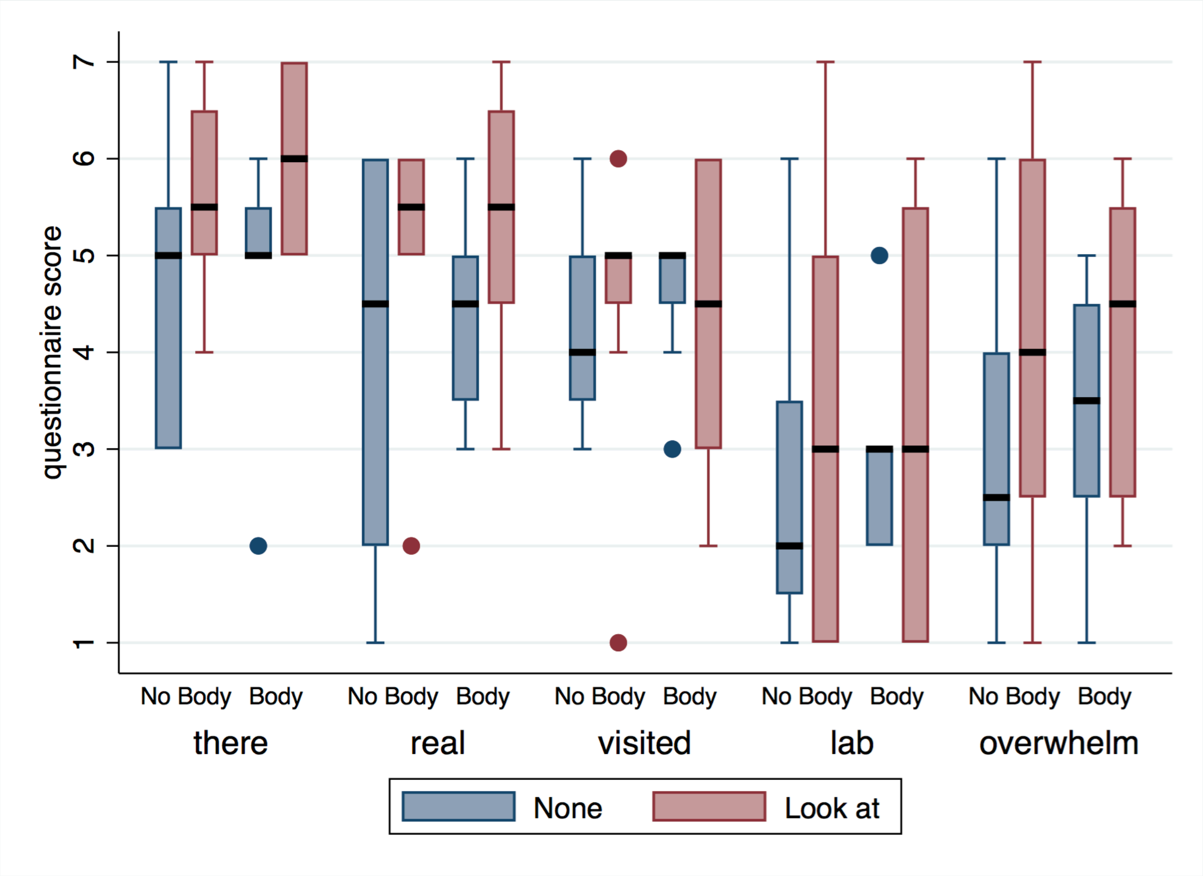

Figure S1 – Box plots of questionnaire responses on Place Illusion (Table 1). The thick horizontal lines are the medians, and the boxes the interquartile ranges (IQR). The whiskers extend from max(min value,lower quartile – 1.5*IQR) to min(max value, upper quartile + 1.5*IQR). Values outside of these ranges are shown as individual points.

Figure S2 shows the box plots for the questions on Plausibility of the situation. The medians are above the mid-point, with 6 of the 10 scores greater for Interact than No Interact.

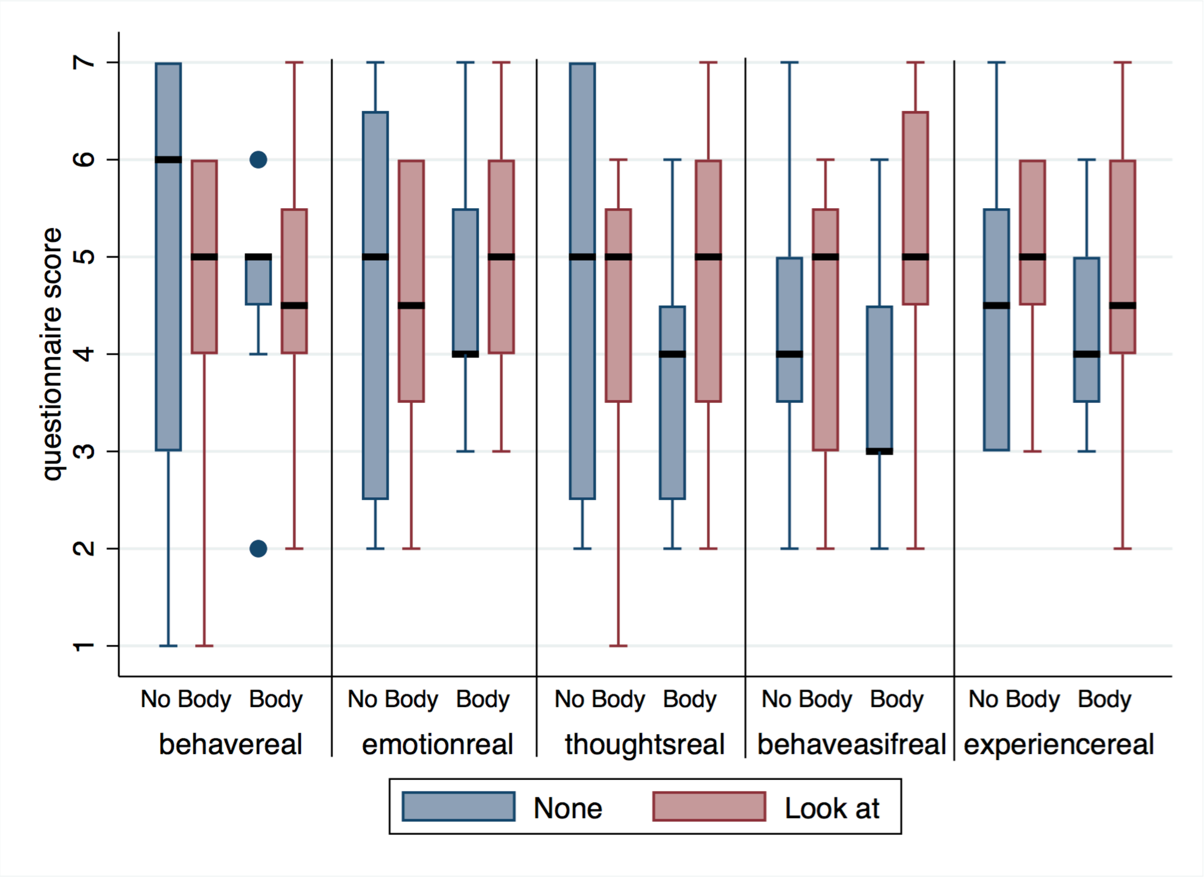

Figure S2 – Box plots of questionnaire responses on Plausibility of the situation (Table 1).

Figure S3 shows that the virtual people seemed real more in the case of the Body and Interact conditions than in the remaining conditions (peoplereal). The same is true for *emotionpeople* and *thoughtspeople*. Overall apart from *physicalpeople*, the scores tend to be higher for the Interact than the No Interact conditions, and there is little visible influence of the Embodiment. In fact the medians are greater for Interact than No Interact for all but two of the scores, where they are equal.

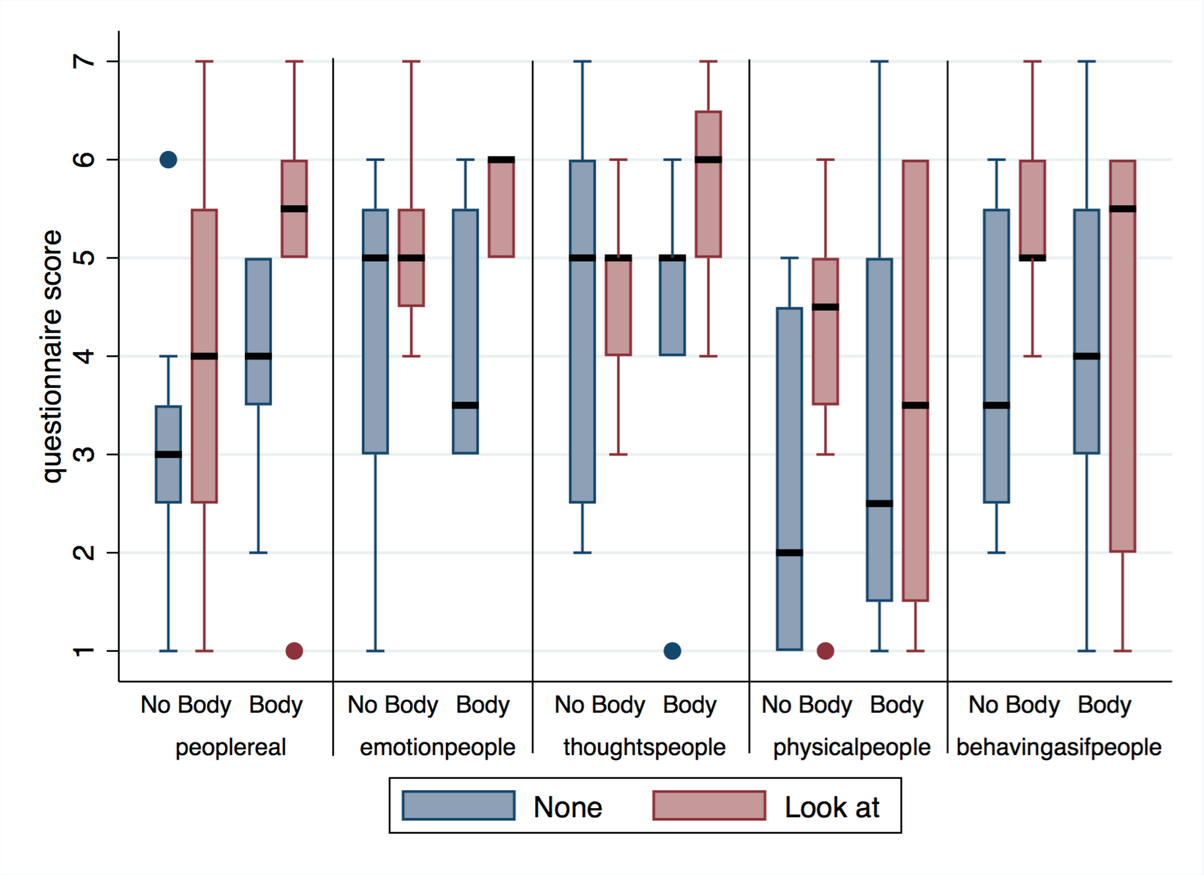

Figure S3 – Box plots of questionnaire responses on Plausibility of the virtual people (Table 1).

Figure S4 shows strange results where *mybody* is highest for the Body with no responsiveness, and also *otherbody* with no responsiveness but in the body condition is as high as *mybody* with a body and no responsiveness. These response variables were not considered further in the paper since they do not make sense. It is quite possible that this question was misinterpreted: "Other Body: During the experience I felt that the virtual body belonged to someone else". For participants who were hardly aware that they had a virtual body they could have interpreted this as relating to the other virtual bodies that they saw.

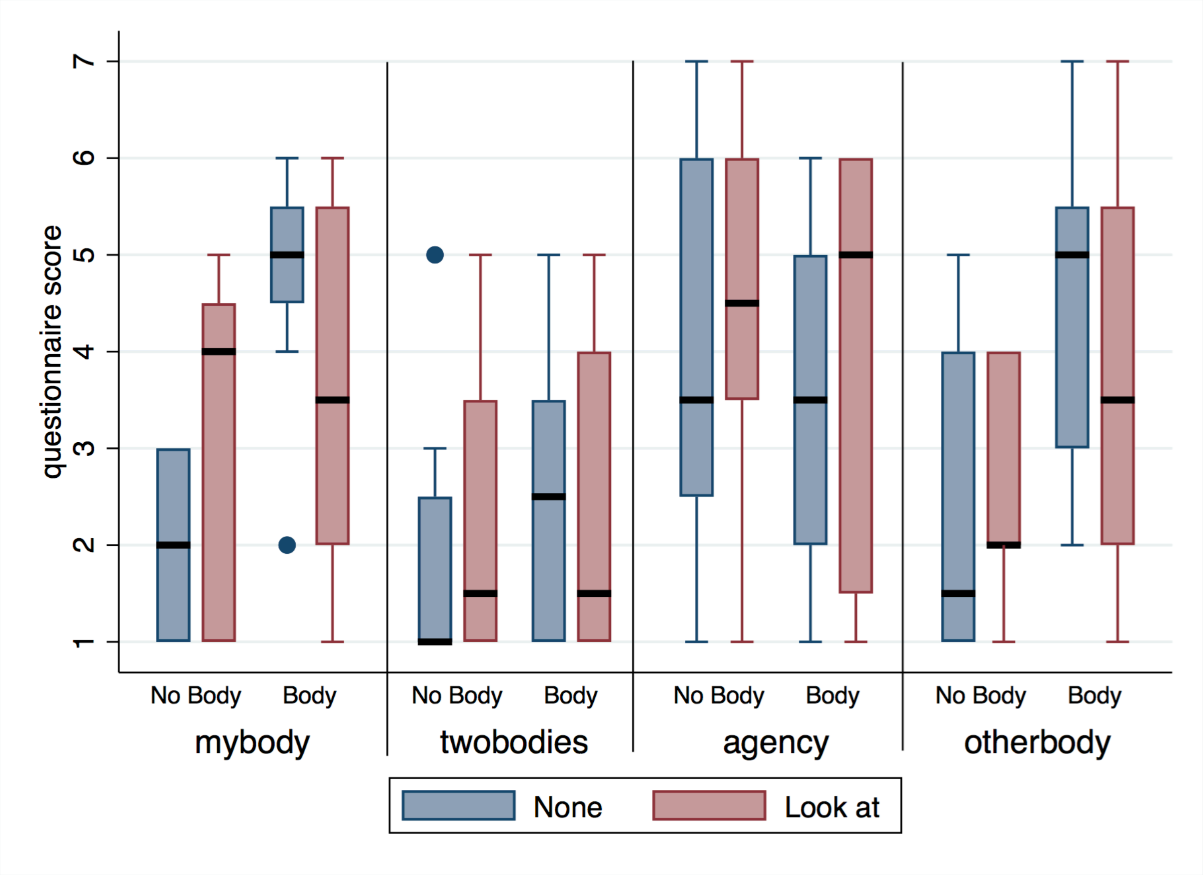

Figure S4 – Box plots of questionnaire responses on body ownership and agency (Table 1).
